# Therapist-Level Moderators of Patient-Therapist Match Effectiveness in Community Psychotherapy

**DOI:** 10.1007/s10488-024-01360-8

**Published:** 2024-04-02

**Authors:** Alice E. Coyne, Michael J. Constantino, James F. Boswell, Averi N. Gaines, David R. Kraus

**Affiliations:** 1 Department of Psychology, American University, 4400 Massachusetts Ave NW, Washington, DC 20016, USA; 2 Department of Psychological and Brain Sciences, University of Massachusetts, Amherst, MA USA; 3 Department of Psychology, University at Albany, State University of New York, Albany, NY USA; 4 Outcome Referrals Inc, Framingham, MA USA

**Keywords:** Patient-therapist matching, Treatment personalization, Therapist-level moderators, Measurement-based care, Naturalistic psychotherapy

## Abstract

Based on patient-reported outcomes data analyzed at the provider level, there is evidence that psychotherapists can possess effectiveness strengths and weaknesses when treating patients with different presenting concerns. These within-therapist differences hold promise for personalizing care by prospectively matching patients to therapists’ historical effectiveness strengths. In a double-masked randomized controlled trial (RCT; NCT02990000), such matching outperformed pragmatically determined usual case assignment—which leaves personalized, measurement-based matching to chance—in naturalistic outpatient psychotherapy (Constantino et al., JAMA Psychiatry 78:960–969, 2021). Demonstrating that personalization can be even more precise, some research has demonstrated that the strength of this positive match effect was moderated by certain patient characteristics. Notably, though, it could also be that matching is especially important for some *therapists* to achieve more effective outcomes. Examining this novel question, the present study drew on the Constantino et al. (JAMA Psychiatry 78:960–969, 2021) trial data to explore three therapist-level moderators of matching: (a) effectiveness “spread” (i.e., greater performance variability across patients’ presenting problem domains), (b) overestimation of their measurement-based and problem-specific effectiveness, and (c) the frequency with which they use patient-reported routine outcomes monitoring in their practice. Patients were 206 adults, randomized to the match or control condition, treated by 40 therapists who were crossed over conditions. The therapist variables were assessed at the trial’s baseline and patients’ symptomatic/functional impairment and global distress were assessed regularly up to 16 weeks of treatment. Hierarchical linear models revealed that only therapist effectiveness spread significantly moderated the match effect for the global distress outcome; for therapists with more spread, the match effect was more pronounced, whereas the match effect was minimal for therapists with less effectiveness spread. Notably, two therapist-level covariates unexpectedly emerged as significant moderators for the symptomatic/functional impairment outcome; for clinicians who consistently treated patients with higher versus lower average severity levels and who relatedly treated a higher proportion of patients with *primary* presenting problems of substance misuse or violence, the beneficial match effect was even stronger. Thus, measurement-based matching may be especially potent for therapists with more variable effectiveness across problem domains, and who consistently treat patients with more severe presenting concerns or with particular primary problems, which provides further precision in conceptualizing personalized care.

**Supplementary Information:**

The online version contains supplementary material available at 10.1007/s10488-024-01360-8.

## Introduction

The analysis of patient-reported outcomes data at the provider level has revealed various types of psychotherapist effectiveness differences (Wampold & Owen, 2021). For one type, which relies on measuring patient outcomes multidimensionally, a growing literature has demonstrated that many community-based psychotherapists possess—across the average patient on their caseload—effectiveness strengths and weaknesses in treating different types of specific and dimensionally rated mental health concerns, even when controlling for patient case-mix factors known to influence treatment outcomes (e.g., Kraus et al., 2011, 2016). For any provider, these so-called *within-therapist effects* can form a multidimensional performance profile across psychometrically distinct problem domains (see Constantino et al., 2021; Coyne, 2024).

For example, whereas Therapist X may be consistently and exceptionally *effective* when treating their average patient with primary anxiety or quality of life deficits (i.e., these patients show substantial improvement or change beyond an empirically predicted level), they may also be reliably *ineffective* when treating their average patient with primary substance misuse or sleep difficulties (i.e., these patients show little-to-no improvement, a failure to improve to an empirically predicted level, or even meaningful deterioration over treatment). Therapist X could also be reliably *neutral*, or performing as expected, when treating their average patient with depression (i.e., these patients show some improvement or change near, but not beyond or below, an empirically predicted level; see Constantino et al., 2021 and the Method section in this paper for a further explication of such performance classifications). Moreover, when multidimensional outcomes data are accumulated across separate cohorts of patients, such problem-specific, within-therapist effectiveness differences appear to be stable over time (Kraus et al., 2016).

Unfortunately, to date, most therapists are unaware of their problem-specific measurement-based effectiveness profiles, either because they are not routinely collecting outcomes data in their practice, or they are not analyzing these data systematically at the caseload level. What may be even more problematic is that when asked to judge their own effectiveness, therapists appear to be inaccurate—often overestimating their general strengths and underestimating their general weaknesses when compared to their peers (Walfish et al., 2012), and overestimating their problem-specific strengths and underestimating their problem-specific weaknesses when compared to their own actual measurement-based performances (Constantino et al., 2023). Arguably, such overconfidence bias contributes to a notable public health concern when considering that directories of therapist expertise—on which patients and referrers often rely when searching for a provider—largely reflect these flawed, non-measurement-based self-assessments (i.e., therapists simply check boxes for the types of mental health concerns they treat and for which they presumably have some expertise). Thus, in everyday practice, there is a meaningfully high probability that a patient will be referred to a therapist whom they believe is highly effective in treating their primary problem, when in actuality that therapist may be empirically neutral,^1^ ineffective, or even harmful in this domain. To combat this issue, there would seem to be promise in establishing therapists’ measurement-determined and problem-specific effectiveness profiles and purposefully harnessing these data to facilitate more effective personalized care through purposefully ‘playing to’ providers’ known strengths and ‘playing away from’ from their known weaknesses (Boswell et al., 2016; Constantino & Muir, 2024).

Testing one version of this notion, a recent study (Constantino et al., 2021) first established therapists’ historical effectiveness profiles based on patient-reported routine outcomes data that had been collected for years in a community mental health care network. Specifically, patients completed at pretreatment and follow up the Treatment Outcome Package (TOP; Kraus et al., 2005), which dimensionally assesses the following 12 domains of symptomatic/functional impairment: depression, quality of life, mania, panic/anxiety, psychosis, substance misuse, social conflict, sexual functioning, sleep, suicidality, violence, and work functioning (additional TOP details are presented in the subsequent Method section). As these patient-reported outcomes data accumulated over time, they also provided de facto information on each therapist who had seen enough patients (at least 15) to establish a reliable performance profile across these 12 TOP domains.

Next, a double-masked randomized controlled trial (RCT) demonstrated that patients who were prospectively matched to therapists with empirically determined historical (i.e., pretrial) effectiveness strengths in treating the patient’s most salient problem(s) had significantly better outcomes both in terms of symptomatic/functional impairment (i.e., the TOP total score, which reflected the mean of the 12 domain-specific *z* scores) and global distress (i.e., the total score for the Symptom Checklist-10 [SCL-10]; Rosen et al., 2000) across up to 16 weeks of naturalistically administered outpatient psychotherapy compared to patients who were assigned to therapists through usual pragmatic means (e.g., therapist self-reported specialty, schedule compatibility, insurance acceptance) and for which empirical matching was left purely to chance (Constantino et al., 2021). Put differently, and spotlighting the within-therapist level of analysis, a given trial therapist had better outcomes to a medium-to-large degree (*d*s = 0.75 and 0.50 for the TOP and SCL-10, respectively) when treating their matched versus case-assignment-as-usual (CAU) patients. Notably, strengths-based matching had multiple levels (according to the aforementioned tripartite, TOP-based classification scheme of effective, neutral, and ineffective). Briefly, at the highest level, a therapist had been classified as effective in treating the patient’s three most elevated problem areas. At the lowest level, they were classified as neutral in treating the patient’s three most salient problems, meaning that while their average patient in these domains did not exceed algorithm-predicted improvement levels, they *did* improve to the expected degree (i.e., they were not ineffective or harmful; the additional match levels are described fully in the subsequent Method section). Notably, Constantino et al. (2021) reported that the main effect of matching differed by level on the TOP outcome. As would be expected, the largest effect of match versus no match (*d* = 1.25) occurred at the highest level; however, even at the lowest level, the effect was still medium-to-large (*d* = 0.75).

Moreover, underscoring that personalizing care to the provider can be even more precise, the beneficial overall effect of matching (across the match levels) was especially pronounced for patients with more severe pretreatment problems and, distinctly, for those who had historically underrepresented racial/ethnic identities (Boswell et al., 2022). This patient-level heterogeneity of matching is akin to the aptitude by *treatment* interaction effect that has long been the flagship for personalization research in psychotherapy (e.g., Coyne et al., 2022; Snow, 1991). In this case, though, with treatment being unmanipulated in the Constantino et al. (2021) trial, the personalization effects were aptitude (or perhaps more accurately, “characteristic”) by *case-assignment* interactions.

Importantly, however, the pursuit of precision care need not stop at such patient-level moderators of problem-centered patient-therapist matching. Although this is a promising step in expanding the precise utility of measurement-based care, it is also conceivable that matching of this type may be especially important for some *therapists* to achieve more effective outcomes. Supporting this contention in a general sense, a subsidiary analysis in Constantino et al.’s (2021) study showed that the effect of matching varied significantly among therapists; that is, matching was more strongly beneficial for some therapists than others. Although there is no existing work on the therapist-level factors that explain such differential effects of prospectively matching patients to therapists’ problem-specific strengths, multiple factors could have conceptual and clinical relevance and therefore represent a good exploratory starting point.

First, it is plausible that for therapists in the Constantino et al. (2021) trial who historically showed greater performance variability across patients’ presenting problem domains (i.e., they had at least one problem domain for which they were classified as effective and at least one problem domain for which they were classified as ineffective), it was especially important for their patients to be empirically matched to an effectiveness area (or at least away from ineffectiveness or harm). The rationale supporting this possibility is that possessing at least this minimal level of such therapist effectiveness “spread” would have rendered it more likely that a patient’s assignment to the match condition resulted in being paired with an exceptionally effective provider (vs. neutral only—the aforementioned lowest match level). On the other side of the coin, the presence of some effectiveness spread would have rendered it more likely that a patient’s assignment to the CAU condition resulted, by chance, in being paired with a personally ineffective provider.

Second, it is possible that the match effect in the Constantino et al. (2021) trial was especially potent for therapists who had more inaccurate perceptions of their own pretrial, domain-specific performance (i.e., they overestimated their performance as effective in domains in which measurement actually classified them as neutral or ineffective). The rationale supporting this possibility is that such overconfidence bias may have increased the likelihood in the CAU condition of a chance-based poor fit to a personally ineffective provider given that therapists’ (often flawed) self-reported specialty was one pragmatic means that often drove usual case assignments. Finally, it is plausible that for therapists who reported less frequent use of patient-reported routine outcomes monitoring (ROM) in their everyday practice, it was especially important for their patients to be empirically matched to an effectiveness area (or at least away from ineffectiveness or harm). The rationale for this possibility is that such a professional position could indicate a greater reliance on flawed clinical judgement (Constantino et al., 2023; Walfish et al., 2012), as opposed to measurement-informed care, which could again render patients in the CAU condition at greater risk for poorer outcomes with that provider (and which, conversely, could again render data-informed case assignments an important contributor to that therapist’s effectiveness).

The present study drew on data from the Constantino et al. (2021) trial to examine whether one or more of the three aforementioned therapist variables moderated the overall match effect on each of the two outcomes of symptomatic/functional impairment and global distress. Given the lack of prior research on therapist-level moderation of matching, all analyses were exploratory.

## Method

### Dataset Overview

As noted, Constantino et al.’s (2021) RCT tested the effectiveness of matching patients to therapists’ problem-specific strengths prior to receiving unmanipulated outpatient therapy. The trial took place across six clinics embedded in a large private community mental health network in Cleveland, OH, and posttreatment was operationalized as the time at which a patient terminated treatment up to a maximum of 16 weeks after baseline. To reiterate the main match effect on the outcomes, hierarchical linear models (HLMs) demonstrated that matched versus CAU patients experienced significantly greater weekly improvement in both their general symptomatic/functional impairment, as per the TOP total score (*γ*_110_ = − 0.03; 95% CI: − 0.05 to − 0.01), and their global psychological distress, as per the SCL-10 total score (*γ*_110_ = − 0.16; 95% CI: − 0.30, − 0.02). The size of the match effect was medium-to-large for symptomatic/functional impairment (*d* = 0.75) and medium for global psychological distress (*d* = 0.50). (As therapists were crossed over conditions, the main effect was a within-therapist effect; a given trial therapist had better outcomes when treating their matched versus CAU patients.) The present study explored whether this same main effect on outcomes was moderated by the three baseline therapist-level variables.

### Participants

#### Therapists

The Constantino et al. (2021) trial included 48 therapists. Given the present study’s focus on therapist-level moderators of matching, we included a subsample of 40 therapists who (a) treated more than one study patient (*M* = 5.15; range = 2–11) and (b) had pretrial performance profiles that allowed them to treat patients in both the match and CAU conditions (as discussed more fully in the subsequent Match Manipulation and Treatment section). Demographically, these subsample therapists averaged 49.43 years (*SD* = 14.10), and the majority identified as White (78.0%) and as a woman (68.3%). Professionally, the majority of providers had received master’s-level training (63.4%) and reported an average of 16.03 years (*SD* = 12.02) of post-degree experience. The eight therapists excluded from the present subsample did not differ from the remaining therapists on any demographic or professional characteristics (all *p*s > 0.05).

#### Patients

Eligible patients for the Constantino et al. (2021) trial were adults (aged 18–70) who naturally presented for outpatient therapy and made all of their own mental health care decisions. Although 218 patients were randomly assigned to either the match (*n* = 99) or CAU (*n* = 119) conditions, given the present study’s aforementioned *therapist* inclusion criteria, the effective patient subsample included only the 206 individuals (*n* = 98 match; *n* = 108 CAU) treated by the 40 subsample therapists. For this patient subsample, Table 1 shows the demographic and clinical characteristics by case-assignment condition. Importantly, the 12 patients excluded from the present subsample did not differ from the remaining patients on any demographic or clinical characteristics (all *p*s > 0.05). Additionally, within the present patient subsample, there were no significant between-condition differences on any such variables (all *p*s > 0.05). Across both conditions, the following percentages reflect subsample patients’ most elevated (severe) presenting TOP domain: quality of life deficits (21.4%), depression (19.9%), substance misuse (18.0%), panic/anxiety (9.7%), social functioning (5.8%), suicidal ideation (6.3%), sleep (5.8%), sexual functioning (4.9%), psychosis (3.9%), work functioning (1.9%), violence (1.9%), and mania (0.5%).^2^ For these dimensionally rated primary problems, the average severity level was approximately 4 *SD*s greater than a non-treatment-seeking population (average *z* score = 4.04; *SD* = 2.76). Thus, in terms of primary presenting problem, this sample was relatively severe. However, for comparison purposes, with regard to *overall* symptomatic/functional impairment, the sample demonstrated more moderate severity, with an average TOP total score approximately 1 *SD* greater than a non-treatment-seeking population (average *z* score = 0.96; *SD* = 0.88).

**Table 1:** Patient baseline demographic and clinical characteristics by condition (N = 206)

	CAU (*n* = 108)			Match (*n* = 98)		
**Variables**	*M*	*SD*	*n* (%)	*M*	*SD*	*n* (%)
Age	34.52	11.67		33.09	10.50	
Sex						
Female			73 (67.6)			65 (66.3)
Male			35 (32.4)			33 (33.7)
Race/ethnicity						
Caucasian/White			96 (88.9)			86 (87.8)
Hispanic/Latino			3 (2.8)			3 (3.1)
African American/Black			5 (4.6)			7 (7.1)
Asian			2 (1.9)			2 (2.0)
Other			2 (1.9)			0 (0.0)
Sexual orientation						
Heterosexual			87 (80.6)			88 (89.8)
Bisexual			4 (3.7)			3 (3.1)
Gay or lesbian			9 (8.3)			6 (6.1)
Not sure			5 (4.6)			0 (0.0)
Missing			3 (2.8)			1 (1.0)
Annual household income						
Less than 20,000			6 (5.6)			7 (7.1)
20,000–40,00			10 (9.3)			10 (10.2)
40,000–75,000			34 (31.5)			28(28.6)
75,000–100,000			20 (18.5)			24 (24.5)
100,000 or more			35 (32.4)			28 (28.6)
Missing			3 (2.8)			1 (1.0)
Education						
High school or less			14 (13.0)			18 (18.4)
Business or trade school			6 (5.6)			8 (8.2)
2-year college			10 (9.3)			13 (13.3)
4-year college			38 (35.2)			29 (29.6)
Masters or doctorate			31 (28.7)			22 (22.4)
Missing			9 (8.3)			8 (8.4)
Marital status						
Single			49 (45.4)			45 (45.9)
Married/cohabiting			49 (45.4)			43 (43.9)
Divorced/widowed/separated			7 (6.5)			9 (9.2)
Missing			3 (2.8)			1 (1.0)
Previous therapists/courses of therapy^a^	1.76	1.92		1.56	1.51	
On psychiatric medication?						
Yes			27 (25.0)			23 (23.5)
No			40 (37.0)			46 (46.9)
Missing^b^			41 (38.0)			29 (29.6)
Baseline TOP global symptomatic/functional impairment *(M* of the 12 domain-specific *z* scores)	0.89	0.80		1.05	0.97	
Baseline SCL-10 global distress	15.60	8.13		16.12	7.77	

CAU, Case assignment as usual; *M*, mean; *SD*, standard deviation; TOP, Treatment Outcome Package; SCL-10, Symptom Checklist-10. ^a^Note that *n* = 201 for this variable due to missing data. ^b^The total sample size for the psychiatric medication item is 136 because of a technological error during data collection

### Match Manipulation and Treatment

As noted, the match system in the Constantino et al. (2021) trial was driven by therapists’ historical effectiveness profiles across the TOP’s 12 dimensionally rated domains of symptomatic/functional impairment. To establish these profiles, the researchers compared each therapist’s patient-reported pre-posttreatment TOP data across at least 15 total pretrial cases (*M* = 28.48; *SD* = 3.00) with TOP data from a large community outpatient reference sample for which case-mix-adjusted, normative (expected) rates of change had been established (see Kraus et al., 2011, 2016). Based on these comparisons, and as previously stated, therapists were classified for each TOP domain as effective (a relative strength), neutral, or ineffective (a relative weakness). To reiterate, an effective classification indicated that the therapist’s average patient in a given domain reliably exceeded their personally predicted/expected rate of improvement based on their baseline TOP and case-mix profile (i.e., the therapist’s performance in this domain was exceptional). A neutral classification signified that the therapist’s average patient in a given domain reliably changed to about the expected degree for their baseline profile (i.e., the therapist’s performance in this domain was generally positive, though not exceptional). Finally, an ineffective classification indicated that the therapist’s average patient in a given domain fell short of their personally expected rate of improvement (i.e., the therapist underperformed in this domain and may have even been harmful). Once these multidimensional classifications were established at the trial’s baseline, the match system could be implemented for the manipulated case assignments with trial patients.

Patients randomized to the match condition in the Constantino et al. (2021) trial were assigned to an empirically matched therapist at one of five levels.^3^ As stated previously, level 1 was the highest level of matching for which the therapist had been historically classified as effective in treating the patient’s three most elevated TOP domains, and was also not historically ineffective on any of the other nine domains (at this level of matching in the main trial, there were 9.1% of match patients and 1.7% of CAU patients). At level 2, the therapist was historically effective on the patient’s single-most elevated TOP domain, and not ineffective on any of the other 11 domains (at this level of matching, there were 28.3% of match patients and 14.3% of CAU patients). At level 3, the therapist was effective on the patient’s three most elevated TOP domains, but was also ineffective on one or more of the other 11 domains (at this level of matching, there were no patients in either condition). At level 4, the therapist was effective on the patient’s single-most elevated TOP domain, but was also ineffective on one or more of the other 11 domains (at this level of matching, there were 4.0% of match patients and 1.7% of CAU patients). Finally, at level 5 (the lowest match level), the therapist was not classified as effective on any of the patient’s three most elevated TOP domains, but was also not ineffective on any of the 12 TOP domains (i.e., they were classified as neutral across the board, meaning that this level of match essentially meant avoiding historically harmful providers; at this level of matching, there were 58.6% of match patients and 46.2% of CAU patients^4^).

Descriptively, 43 of the 48 trial therapists were eligible to see patients in the match condition, either because they were classified as effective in at least one TOP domain or because they were classified as neutral in all 12 TOP domains, the latter of which allowed for level-5 matching (the other five therapists saw patients only in the CAU condition and, as noted, were not included in the present study; Constantino et al., 2021). As per Constantino et al. (2021), therapists had a mean of 1.56 (*SD* = 1.66) domains in which they were classified as effective and 0.96 (*SD* = 1.65) domains in which they were classified as ineffective. No therapist had more than six such strengths, with most having either zero (35%), one (23%), or two (17%). Additionally, no therapist had more than eight such weaknesses, with most having either zero (56%), one (21%), or two (15%).

As noted, patients randomized to the CAU condition were assigned to therapists pragmatically, based on factors like therapist’s self-reported areas of expertise, roster openings, office location, etc. (Constantino et al., 2021). As also stated previously, for this ecologically valid comparator, patients could still be matched by chance to a provider at any of the five levels. Importantly, though, and as expected, significantly more match versus CAU patients were matched at higher levels (see Constantino et al., 2021, for additional manipulation check details). Finally, after the double-masked case assignment, patients were treated as usual (i.e., the research team had no influence over how therapists treated their cases). Therapists did rate at baseline (on a scale from 0 [*not at all*] to 6 [*very much*]) the degree to which various theoretical orientations influenced their practice. These ratings were as follows: cognitive-behavioral (*M* = 5.19; *SD* = 1.05), integrative (*M* = 4.31; *SD* = 1.57), interpersonal (*M* = 3.91; *SD* = 1.56), humanistic/experiential (*M* = 3.31; *SD* = 1.66); systems (*M* = 2.98; *SD* = 1.35), and psychodynamic/psychoanalytic (*M* = 2.12; *SD* = 1.74). Additionally, within the study’s aforementioned 16-week post-baseline design constraint, patients’ average treatment length was 11.49 weeks (*SD* = 6.10), which translated to an average of 5.70 sessions (*SD* = 3.26).

### Measures

#### Therapist Effectiveness Spread

For the first moderator variable, we used the aforementioned therapist domain-specific effectiveness classifications to generate a dichotomous index of effectiveness spread. Specifically, therapists were classified as having high effectiveness spread (1) if they had at least one domain for which they were classified as effective and at least one domain for which they were classified as ineffective. All other patterns of effectiveness strengths and weaknesses (i.e., no strengths or weaknesses, only strengths, or only weaknesses) were classified as having low effectiveness spread (0). We chose this cutoff because, as noted previously, therapists in the Constantino et al. (2021) trial averaged about 1–2 strengths and 0–1 weaknesses.

#### Therapist Overestimation of their Problem-Specific Effectiveness

For the second moderator variable, we used multiple sources of information to create a count variable. First, therapists completed the trial-specific Therapist Perceived Strengths (TPS) questionnaire (Constantino et al., 2021, 2023), which was designed to correspond to effectiveness classifications for each of the 12 TOP domains. Specifically, therapists rated how effective they believed they were in treating these presenting concerns. Sample items included: “In treating my clients’ symptoms of [depression], I would say that I am …”; “In improving my clients’ [panic/anxiety], I would say that I am …”. The scale was: 1 (*always ineffective*), 2 (*usually ineffective*), 3 (*sometimes ineffective*), 4 (*inconsistently ineffective*), 5 (*sometimes effective*), 6 (*usually effective*), 7 (*always effective*). To match the tripartite measurement-based effectiveness classification used in the match system, responses of 1 and 2 were collapsed into ineffective; responses of 3, 4, and 5 were collapsed into neutral; and responses of 6 and 7 were collapsed into effective. Second, we compared therapists’ self-perceived effectiveness categories with their measurement-based effectiveness classifications in order to derive an *overestimation* variable—that is, the number of TOP outcome domains (ranging from 0 to 12) for which therapists’ self-perceived effectiveness categorization was higher than their measurement-based effectiveness classification (e.g., therapists saw themselves as effective in treating a given problem when they were actually historically neutral or ineffective).

#### Therapist Frequency of ROM Usage

For the third moderator variable, therapists completed a trial-specific item embedded in the TPS, which asked them how often they used ROM as a resource in their clinical practice. The scale ranged from 1 (*never use/seek this)* to 5 (*always use/seek this).*

#### Patient Mental Health Symptoms/Functioning

For one outcome variable, we used the same index as the Constantino et al. (2021) trial—general symptomatic/functional impairment as per the TOP (Kraus et al., 2005). This measure includes 58 total items that dimensionally assess the 12 problem domains outlined previously. The scale ranges from 0 (*none*) to 5 (*all*) to capture how much time over the past two weeks the person has experienced a given concern that loads onto one of the domains. For analysis and ease of interpretation, total scores of each of the TOP domains are transformed into *z* scores that reflect *SD* units relative to the general, non-treatment-seeking population mean, with higher scores indicating more impairment (e.g., a score of 2 on the depression scale would reflect a depression level that is 2 *SD*s higher than the general population). The TOP subscales have excellent factor structure, good internal consistency, and good test–retest reliability. The global symptomatic/functional impairment index (TOP total score) represents the mean of the 12 domain-specific *z* scores. This score has been shown to have excellent reliability, convergent validity, and change sensitivity (Kraus et al., 2005; Zack et al., 2015).

#### Patient Global Psychological Distress

For the second outcome variable, we again used the same index as the Constantino et al. (2021) trial—global psychological distress as per the SCL-10 (Rosen et al., 2000). This brief measure includes 10 items rated from 0 (*not at all*) to 4 (*extremely*) that can be summed to create a total score (theoretical range = 0–40), with higher scores indicating greater distress. The SCL-10 has demonstrated good reliability, change sensitivity, and convergent validity with other measure of global distress, including the global severity index from the original long-form version of this scale (i.e., the SCL-90; Rosen et al., 2000).

### Procedure

Within the aforementioned community mental health network, therapist recruitment for the Constantino et al. (2021) trial occurred between August 2017 and January 2019. Potential participants were informed they could participate in a study examining the effectiveness of various referral procedures (about which neither they nor their patients would be aware) and for which there would be no influence on the usual treatment they would administer. Interested therapists were directed to an online consent form and baseline survey, which included a demographic and practice characteristics form and the TPS—the latter of which allowed us to derive two of the therapist-level moderator variables: overestimation of their problem-specific effectiveness and frequency of ROM usage. Additionally, these clinicians’ baseline performance profiles were available to derive the moderator variable of therapist effectiveness spread.

Within the same community mental health network, patient recruitment for the Constantino et al. (2021) trial occurred between November 2017 and April 2019. At intake, potential participants were informed they could participate in a study examining the effectiveness of various referral procedures (about which neither they nor their therapist would be aware) and for which there would be no influence on the usual treatment they would receive. Interested patients were directed to an online consent form and baseline survey, which included the baseline TOP administration that primed the match system and a baseline measures packet that included the SCL-10. Consenting patients were then randomly assigned to case-assignment condition. Pertinent to this study, patients also completed the TOP and SCL-10 every other week during treatment and at their personal posttreatment. A university institutional review board approved the trial, which was preregistered at ClinicalTrials.gov (identifier: NCT02990000), and subsequent analysis of deidentified data.

### Data Analyses

We first calculated descriptive statistics and examined the distributions of all continuous study variables to determine if they were acceptably normally distributed (skewness >  − 2 and < 2). Any variables that were not normally distributed were transformed. Additionally, given our interest in *therapist*-level moderators of the match effect, we descriptively investigated whether there were caseload-level differences in the types of patients each therapist treated that could influence the results. First, we examined whether there was any indication that certain therapists were more likely to treat patients with particular presenting problems. If any such instances were detected (either due to chance or the case assignment manipulation), we controlled in our primary analyses for these differences in the proportion of cases seen by each therapist with a given presenting problem. Second, and somewhat relatedly, because it also seemed plausible that patients with certain presenting problems could be more likely to have higher impairment/distress, we descriptively and inferentially (using Pearson’s correlations) examined whether the caseload-level proportion of each of the different presenting problems was associated with caseload-level differences in overall presenting severity. If such an association was detected, we added it as a covariate.

To test our research questions, we used the HLM program (Raudenbush & Bryk, 2002) to examine the three therapist-level variables (i.e., effectiveness spread, overestimation of problem-specific effectiveness, and frequency of ROM usage) as moderators of the patient-level match effect using a random coefficient prediction framework for testing cross-level interactions (Preacher et al., 2016). More specifically, we fit three (one for each moderator) 3-level models for each of the two outcome variables that included within-patient outcome change over time at level 1, between-patient/within-therapist outcome differences at level 2, and between-therapist outcome differences at level 3. At level 1, consistent with the primary outcomes paper for this trial (Constantino et al., 2021), we fit a linear change trajectory to each patient’s outcome scores.^5^ Time was coded in weeks and centered at week 17 (i.e., 16 treatment weeks plus 1 baseline week) so that the intercept represented each patients’ level of impairment/distress at the end of the study period (hereafter termed “posttreatment”). At level 2, we included match condition (CAU = 0; match = 1) as a predictor of between-patient (within-therapist) differences in both the intercept (i.e., posttreatment impairment/distress level) and slope (i.e., rate of weekly outcome change over treatment). Also at this level, random effects allowed the intercept and slope to vary across patients. At level 3, given our interest in testing cross-level (patient factor x therapist factor) interactions, we included random effects that allowed the patient-level match-outcome associations to vary across therapists (i.e., random slopes). Then, we included the relevant therapist-level moderator variables as a predictor of the within-therapist assignment condition-outcome associations.

To avoid model misspecification, we also included the relevant therapist-level moderator variable as a predictor of therapist-level differences in the intercept and slope (akin to including main effects). For ease of interpretation, all therapist moderator variables were grand-mean centered prior to including them in the models. Additionally, because the aforementioned Boswell et al. (2022) patient-level moderator study found that patient-level baseline impairment severity moderated the match effect, we included within- and between-therapist differences in patients’ presenting distress severity as a covariate at level 2 and level 3, respectively. See the Online Supplement for the full multilevel equation used for each of these models. Across all models, we employed maximum likelihood estimation to address missing data (Raudenbush & Bryk, 2002). This approach allowed us to retain all patients who completed at least one assessment of the outcome variable. Because there were no missing data at the therapist level and all patients completed at least two assessments of the outcome variable (see Constantino et al., 2021), this approach allowed us to retain all subsample participants in our analyses.

## Results

### Preliminary Analyses

All study variables were acceptably normally distributed (skewness >  − 2 and <  + 2). Descriptively, most of the subsample therapists had at least one effectiveness strength and one weakness (*n* = 31; 75.6%) and were therefore coded as having high effectiveness spread. Additionally, overestimation was fairly common; on average, therapists overestimated their own measurement-based effectiveness on 6 out of 12 TOP-based outcome domains (*M* = 6.40; *SD* = 2.58; range = 2 to 12). Finally, on average, therapists reported using ROM “sometimes” to “often” in their practices (*M* = 3.62; *SD* = 1.03).

Regarding instances of therapist-level differences in their proportion of patients with different presenting problems, our descriptive examination revealed one noteworthy pattern. Namely, it appeared that some therapists had a higher or lower than would be expected (based on the 18% base rate in this patient sample) proportion of patients with primary substance misuse (caseload-level proportion of this problem ranged from 0 to 100%). Specifically, 6 therapists (15% of the sample) had caseloads with more than double the expected percentage of individuals with primary substance misuse (i.e., > 36%), and 17 therapists (43%) treated 0 individuals with primary substance misuse. Therefore, we controlled for caseload-level percentage of individuals with primary substance misuse in our analyses.

Additionally, our examination of whether caseload-level proportion of certain presenting problems was associated with greater caseload-level severity revealed two potential associations. First, caseload-level proportion of patients with primary substance misuse was significantly (*p*s < 0.001) and positively associated with higher caseload-level severity (*r*s = 0.68 and 0.39 for the baseline TOP and SCL-10 scores, respectively). Second, although violence was an infrequent primary problem (1.9%), the caseload-level proportion of it was significantly associated with higher caseload-level severity as assessed via the TOP (*r* = 0.38, *p* < 0.001), though it was not associated with SCL-10-based caseload-level severity (*r* = 0.17, *p* = 0.304). Given this pattern of results and the relatively low base rate of violence as a primary problem, we created a composite variable that represented the caseload-level proportion of individuals with a primary problem of substance misuse or violence, and we used it as a covariate in all primary analyses (*M* = 0.21; *SD* = 0.26; range = 0.00 to 1.00). Additionally, given the high correlation between this composite variable and caseload-level TOP-based severity (*r* = 0.75, *p* < 0.001), and to avoid problematic collinearity between covariates, we used the baseline SCL-10 scores to generate our within- and between-therapist severity indices in all analyses.

### Primary Analyses

The full results of each moderator model for TOP total score outcome are reported in Table 2. When controlling for within- and between-therapist differences in baseline distress severity and between-therapist differences in the proportion of patients presenting with primary substance misuse or violence, none of the three therapist variables (i.e., effectiveness spread, overestimation of their problem-specific effectiveness, and frequency of ROM usage) moderated the effect of matching on weekly change in symptomatic/functional impairment or level of such impairment at posttreatment (all *p*s > 0.05).

**Table 2:** Therapist-level Moderators of Match Effects on Patient TOP-Based Symptomatic/Functional Impairment (N = 206)

Fixed effects	Spread		Overestimation		ROM Frequency	
	Coef	95% CI	Coef	95% CI	Coef	95% CI
Posttreatment TOP, γ_000_	0.28**	0.10, 0.47	0.35***	0.18, 0.51	0.34***	0.18, 0.51
Moderator, γ_001_	0.32	− 0.09, 0.72	0.01	− 0.05, 0.08	− 0.02	− 0.17, 0.14
Caseload-level severity, γ_002_	0.03	− 0.01, 0.08	0.02	− 0.02, 0.07	0.03	− 0.02, 0.08
Caseload-level SU/V, γ_003_	0.46	− 0.31, 1.24	0.35	− 0.42, 1.13	0.32	− 0.46, 1.10
Match, γ_010_	− 0.28*	− 0.53, − 0.02	− 0.34**	− 0.57, − 0.11	− 0.36**	− 0.60, − 0.13
Match x Moderator, γ_011_	− 0.45	− 1.04, 0.14	0.05	− 0.04, 0.14	− 0.001	− 0.22, 0.22
Match x Caseload severity, γ_012_	− 0.05	− 0.11, 0.02	− 0.04	− 0.11, 0.02	− 0.05	− 0.12, 0.02
Match x Caseload SU/V, γ_013_	− 1.40*	− 2.58, − 0.22	− 1.03	− 2.21, 0.14	− 1.16	− 2.33, 0.01
Patient-level severity, γ_020_	0.04***	0.02, 0.05	0.03***	0.02, 0.05	0.04***	0.02, 0.05
Change in TOP, γ_100_	− 0.04***	− 0.05, − 0.02	− 0.03***	− 0.04, − 0.02	− 0.03***	− 0.04, − 0.02
Moderator, γ_101_	0.02	− 0.01, 0.04	0.0001	− 0.004, 0.004	− 0.004	− 0.01, 0.01
Caseload-level severity, γ_102_	− 0.0007	− 0.003, 0.002	− 0.001	− 0.004, 0.002	− 0.001	− 0.003, 0.002
Caseload-level SU/V, γ_103_	− 0.02	− 0.07, 0.02	− 0.03	− 0.07, 0.02	− 0.03	− 0.08, 0.02
Match, γ_110_	− 0.02*	− 0.03, 0.003	− 0.02**	− 0.04, − 0.01	− 0.02**	− 0.04, − 0.01
Match x Moderator, γ_111_	− 0.02	− 0.06, 0.01	0.003	− 0.003, 0.01	0.003	− 0.01, 0.02
Match x Caseload severity, γ_112_	− 0.01*	− 0.01, − 0.002	− 0.01*	− 0.01, − 0.001	− 0.01*	− 0.01, − 0.002
Match x Caseload SU/V, γ_113_	− 0.10*	− 0.17, − 0.02	− 0.08*	− 0.15, − 0.01	− 0.09*	− 0.16, − 0.01
Patient-level severity, γ_120_	− 0.002***	− 0.003, − 0.001	− 0.002***	− 0.003, − 0.001	− 0.002***	− 0.003, − 0.001

Coef., Coefficient; CI, Credible interval; ES, Effect size; TOP, Treatment Outcome Package. SU/V, Substance misuse and violence as primary problems. *p < .05; **p < .01; ***p < .001

**Fig. 1:**
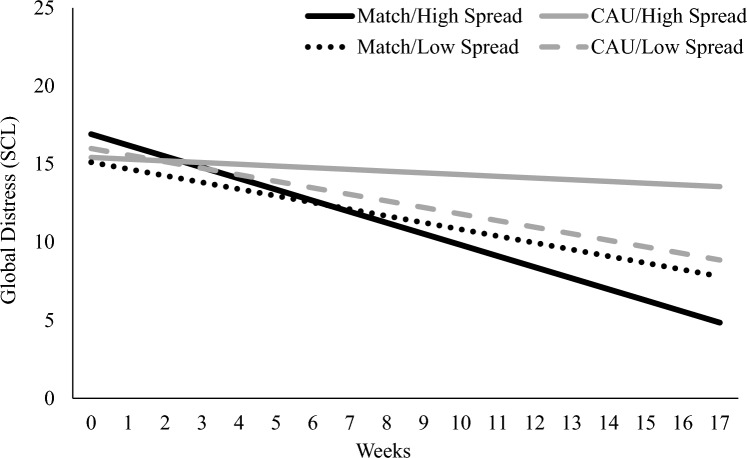
Therapist effectiveness spread as a between-therapist moderator of the within-therapist match effect on global distress. *Note* The outcome variable (depicted on the y-axis) represents the SCL-10 total score (theoretical range = 0–40). Black lines depict average outcomes for different subgroups of patients in the match condition, whereas the CAU condition is represented by gray lines. Solid lines depict average outcomes for therapists with high effectiveness spread and dashed lines represent average outcomes for therapists with low effectiveness spread

The full results of each moderator model for SCL-10 outcome are reported in Table 3. When controlling for all within- and between-therapist covariates, therapist effectiveness spread significantly moderated the effect of matching on weekly change in global distress (*γ*_111_ = − 0.68, *SE* = 0.20, *p* = 0.002) and level of posttreatment global distress (*γ*_011_ = − 10.11, *SE* = 3.43, *p* = 0.006). As depicted in Fig. 1, therapists with high effectiveness spread (i.e., at least 1 strength and 1 weakness) had significantly better outcomes when they treated match versus CAU patients. In contrast, therapists with low effectiveness spread had similar outcomes when they were treating both match and CAU patients. In terms of effect size, among therapists with high effectiveness spread, their average match patient had a posttreatment global distress level that was 1.11 *SD*s lower than their average CAU patient. (Given differences in treatment length across patients, it is worth noting that this difference was moderately sized [*d* = 0.65] by week 11, which was the sample average number of treatment weeks.^6^) In contrast, for therapists with low effectiveness spread, the difference in global distress levels between their average match and CAU patient was never more than small-sized (posttreatment *d* = 0.13). Neither of the other two therapist variables (i.e., overestimation of their problem-specific effectiveness and frequency of ROM usage) moderated the match effect on weekly global distress change or posttreatment global distress level (all *p*s > 0.05; Table 3).

**Table 3:** Therapist-level Moderators of Match Effects on Patient SCL-Based Global Distress (N = 206)

Fixed effects	Spread		Overestimation		ROM frequency	
	Coef	95% CI	Coef	95% CI	Coef	95% CI
Posttreatment SCL-10, γ_000_	8.84***	6.70, 10.97	9.86***	7.91, 11.81	9.87***	7.91, 11.83
Moderator, γ_001_	4.48	− 0.17, 9.14	0.07	− 0.68, 0.83	0.07	− 1.77, 1.91
Caseload-level severity, γ_002_	0.52	0.001, 1.04	0.44	− 0.10, 0.99	0.45	− 0.10, 1.01
Caseload-level SU/V, γ_003_	0.47	− 8.37, 9.31	− 1.50	− 10.56, 7.56	− 1.82	− 10.82, 7.17
Match, γ_010_	− 1.02	− 3.87, 1.83	− 2.76	− 5.53, 0.01	− 3.03*	− 5.91, − 0.14
Match x Moderator, γ_011_	− 10.11**	− 16.84, − 3.38	0.56	− 0.56, 1.68	− 0.25	− 3.00, 2.50
Match x Caseload severity, γ_012_	− 0.28	− 1.05, 0.49	− 0.29	− 1.10, 0.53	− 0.30	− 1.16, 0.57
Match x Caseload SU/V, γ_013_	− 15.39*	− 29.05, − 1.72	− 7.50	− 21.57, 6.57	− 8.60	− 23.00, 5.80
Patient-level severity, γ_020_	0.35***	0.19, 0.51	0.35***	0.19, 0.51	0.35***	0.19, 0.51
Change in SCL-10, γ_100_	− 0.40***	− 0.53, − 0.27	− 0.34***	− 0.45, − 0.22	− 0.34***	− 0.45, − 0.22
Moderator, γ_101_	0.27	− 0.01, 0.54	0.01	− 0.04, 0.05	− 0.003	− 0.11, 0.11
Caseload-level severity, γ_102_	− 0.02	− 0.05, 0.01	− 0.02	− 0.05, 0.01	− 0.02	− 0.05, 0.01
Caseload-level SU/V, γ_103_	0.05	− 0.47, 0.58	− 0.06	− 0.59, 0.48	− 0.08	− 0.61, 0.45
Match, γ_110_	− 0.04	− 0.21, 0.14	− 0.15	− 0.32, 0.02	− 0.17	− 0.34, 0.01
Match x Moderator, γ_111_	− 0.68**	− 1.08, − 0.28	0.03	− 0.04, 0.10	0.0003	− 0.16, 0.16
Match x Caseload severity, γ_112_	− 0.03	− 0.07, 0.02	− 0.03	− 0.08, 0.02	− 0.03	− 0.08, 0.02
Match x Caseload SU/V, γ_113_	− 0.86	− 1.70, − 0.02	− 0.33	− 1.20, 0.53	− 0.41	− 1.29, 0.47
Patient-level severity, γ_120_	− 0.04***	− 0.05, − 0.03	− 0.04***	− 0.05, − 0.02	− 0.04***	− 0.05, − 0.03

Coef., Coefficient; CI, Credible interval; ES, Effect size; SCL-10, Symptom Checklist-10. SU/V, Substance misuse and violence as primary problems. *p < .05; ** *p* < .01; *** *p* < .001

### Ancillary and Sensitivity Analyses

Across the three moderator models for the TOP-based symptomatic/functional impairment outcome, the two therapist-level covariates of caseload-level differences in severity of patients’ presenting global distress and caseload-level differences in the proportion of patients presenting with primary substance misuse or violence unexpectedly emerged as significant moderators of the match effect on weekly symptomatic/functional impairment reduction (see Table 2). Given these results, we fit a final exploratory moderator model. Namely, because a previous study that followed up the Constantino et al. (2021) trial found that *patient*-level severity also moderated the match effect (Boswell et al., 2022), we treated this additional model as a sensitivity analysis to investigate whether the two severity moderator findings and the caseload-level problem type finding were independent. Namely, we simultaneously tested the two cross-level interactions (i.e., match condition x caseload-level severity, match condition x caseload-level proportion of patients presenting with primary substance misuse or violence) while controlling for the patient-level severity x match condition interaction.

Results indicated that the match effect on weekly symptomatic/functional impairment reduction was more pronounced for therapists who treated a higher proportion of patients with primary substance misuse or violence (*γ*_101_ = − 0.09, *SE* = 0.04, *p* = 0.022), though this interactive effect was only a nonsignificant trend for posttreatment symptomatic/functional impairment level (*γ*_011_ = − 1.20, *SE* = 0.60, *p* = 0.052). In terms of effect size, for every 1 *SD* increase in this caseload-level proportion, the match effect on symptomatic/functional impairment reduction became approximately 1.47 × stronger, though the posttreatment match effect remained similarly sized (i.e., 1.01 × stronger; see Supplemental Fig. 1). Similarly, the match effect on weekly symptomatic/functional impairment reduction was also more pronounced for therapists who treated a higher severity caseload (*γ*_101_ = − 0.01, *SE* = 0.002, *p* = 0.008), though this interaction was not significant for posttreatment symptomatic/functional impairment level (*γ*_011_ = − 0.05, *SE* = 0.03, *p* = 0.129). In terms of effect size, for every 1 *SD* increase in caseload-level baseline severity, the match effect on symptomatic/functional impairment reduction became approximately 1.44 × stronger and the posttreatment difference became slightly larger (approximately 1.23x; see Supplemental Fig. 2). Confirming what was previously established (Boswell et al., 2022), the *patient*-level baseline global distress severity moderator was significant for both weekly symptomatic/functional impairment reduction (*γ*_130_ = − 0.002, *SE* = 0.001, *p* = 0.023) and posttreatment symptomatic/functional impairment level (*γ*_030_ = − 0.05, *SE* = 0.1, *p* < 0.001).

As a final sensitivity analysis, we replicated the significant moderator models including only therapists who saw fully crossed caseloads, treating an average of 51% match and 49% CAU cases (*n* = 31 therapists; *n* = 174 patients); that is, despite all 40 therapists in this study being eligible to treat both match and CAU cases, in practice, 9 therapists treated primarily or all match or CAU cases by chance. Results indicated that the match effect remained significantly more pronounced for therapists with versus without effectiveness spread both in terms of weekly change in global distress (*γ*_111_ = − 0.79, *SE* = 0.23, *p* = 0.002) and level of posttreatment global distress (*γ*_011_ = − 11.51, *SE* = 3.81, *p* = 0.005). Similarly, both caseload-level severity (*γ*_111_ = − 0.01, *SE* = 0.003, *p* = 0.008) and proportion of patients with primary substance misuse or violence (*γ*_111_ = − 0.15, *SE* = 0.05, *p* = 0.003) remained significant moderators of the match effect on weekly impairement change. Additionally, a greater proportion of patients with primary substance misuse or violence significantly (vs. the previous trend-level association) magnified the match effect on posttreatment global distress level (*γ*_011_ = − 1.86, *SE* = 0.71, *p* = 0.014).

## Discussion

This study explored whether any of three therapist-level variables (i.e., effectiveness spread, overestimation of problem-specific effectiveness, and frequency of ROM usage) moderated the previously established beneficial effect on patient outcomes (relative to CAU) of prospectively matching adult outpatients to therapists’ historical effectiveness strengths prior to naturalistic therapy. Of these moderators, only therapist effectiveness spread significantly moderated this main effect. Specifically, the match effect on global distress was more pronounced for therapists with high effectiveness spread, whereas the match effect was small and nonsignificant for those with low spread. However, caution is warranted when interpreting this interaction, as it was not significant for the TOP-based symptomatic/functional impairment outcome. Instead, for the TOP-based outcome, two therapist-level covariates emerged as significant moderators; for clinicians who (a) treated trial patients with higher versus lower average presenting severity levels and (b) treated a greater proportion of trial patients with a primary problem of substance misuse or violence, the positive match effect was even stronger.

The finding that a greater number of therapist domain-specific strengths and weaknesses (i.e., effectiveness spread) made matching more beneficial for a given therapist’s outcomes is consistent with the nature of the match system (Constantino et al., 2021). That is, it would make sense that a therapist possessing one or more strengths would have increased the likelihood that a given patient’s assignment to the match condition resulted in being paired with a provider who was exceptionally effective at treating their primary concern(s) (vs. neutral only—the aforementioned lowest match level). Similarly, when therapists possess high spread (and therefore at least one relative weakness) but do not receive matched case assignments, it leaves open the possibility that some of the patients referred to them could have elevated symptoms on a problem for which they are acutally ineffective. Interestingly, the present results seem to be primarily driven by this latter scenario; as depicted in Fig. 1, therapists with high effectiveness spread had the worst outcomes when treating patients in the CAU condition (even relative to therapists with low effectiveness spread). Preliminarily, this suggests that matching may be most beneficial for therapists who possess at least some areas of strength and weakness, because it helps to prevent instances of patients being paired with a personally ineffective provider.

Clinically, this moderator result suggests that the clinicians and care networks may wish to prioritize matching for clinicians with high levels of effectiveness spread, and perhaps especially for clinicians who have one or more areas of relative weakness. In contrast, clincians with more consistent effectiveness across problem domains (which most typically meant neutral performance across most domains) seem to be comparably effective whether they receive matched case assignments or not. Therefore, these clinicians could be regarded as effective generalists who can preserve degrees of freedom within care networks, allowing therapists who have more varied performance profiles (i.e., more strengths, but also more areas of ineffectiveness) to preserve openings for the patients who represent the best matches for their skillsets.

However, it is worth reiterating that this interactive effect and any clinical implications deriving from it should be considered preliminary, as it was only significant for the SCL-based global distress outcome (but not for the TOP-based symptomatic/functional impairment outcome). Although the precise reason for this pattern of results is unknown, there are several possible explanations. First, two covariates emerged as significant moderators for the TOP-based outcome, which could have limited the power we had to detect an additional interactive effect. Second, although this study used the TOP *total score*, it is possible that the multidimensional nature of this measure still influenced the results. That is, based on this study’s preliminary analyses, it appears that a given patient’s total score on the TOP is still influenced by their score on their most elevated domain(s). Relative to a true measure of *global* distress like the SCL, this could have meant that patients’ TOP-based outcomes were primarily influenced by a match or mismatch on their primary problem. Therefore, for the therapist effectiveness spread moderator, instances in which a therapist’s relative weakness(es) was in a non-primary domain for a patient may have had less influence on their TOP-based treatment outcomes than on their global distress outcomes (which may weight different areas of symptoms/functioning more equally). Overall, more research is needed on the match by therapist effectiveness spread interaction, including with more statistical power and different types of outcomes (such as those that truly capture severity in a global sense vs. those that capture severity in a more domain-specific sense).

That there was no moderating influence of the other two therapist variables could simply mean that each has little relevance for understanding the therapist-level variability in the match effect that had emerged in the Constantino et al. (2021) trial. That is, whether therapists are more or less biased in how they view their relative practice strengths or use ROM to a greater or lesser degree, matching patients to providers’ strengths (or at least away from any potential harm at the lowest level) may consistently outperform chance-based matching (based on CAU) to a moderate degree. In parallel, the lack of therapist-level moderation (for these two variables) and the aforementioned mixed results across outcome variables for therapist effectiveness spread, could mean that personalization is indeed best accomplished at the patient level—on which the field has traditionally focused for precision care. That is, irrespective of who the treating clinicians are, if you get patients within a care network to a therapist who has some level of empirical good fit, they will have a better chance of improving than if such matching was left purely to happenstance. Although this interpretation has appeal in highlighting the potential universal benefit of measurement-based matching to therapists’ strengths, it is important to reiterate that it is limited to the moderating variables explored in this study. Thus, it may be that other therapist factors will hold more promise for explaining for whom the match effect on outcome was stronger in the Constantino et al. RCT, as discussed momentarily with regard to the incidental moderators in this study. However, it is also important to highlight potential reasons why the therapist overestimation and frequency of ROM usage explored herein were not significant moderators, but could still serve such a role in other treatment contexts.

First, it is possible that the RCT design undermined the relevance of these variables to the match effect. Namely, all case assignments in the trial were controlled by intake staff. It is conceivable that these moderators would emerge as significant in contexts where therapists are left to make their own case assignments. That is, in such settings, therapists who have an overconfidence bias or are less likely to use ROM data to complement one’s clinical judgment, might be more likely to self-select into poorer matches with patients. Put differently, the non-manipulated case-assignment method in this example would include more risk factors for this hypothetical therapist’s patients to trend toward poor versus accidental good matches. For example, the comparison to level-1 matching in the trial, would be a therapist who generally agreed to see patients with three elevated TOP domains for which that therapist felt they excelled in the absence of data. To the extent that the measurement-based algorithm indicated the therapist was actually ineffective in, say, two of these areas, these patients would be receiving poor matches; hence, this therapist (in light of their overestimation of strengths) would be one for whom measurement-based matching would be especially important for their patients’ outcomes. Future research on matching in settings where therapists make their own decisions for which patients to treat (i.e., a different CAU control condition) is sorely needed.

Unexpectedly, two control variables explained some of the therapist-level heterogeneity in the match effect, despite some of the issues just raised. That therapists’ caseload-level patient baseline severity and tendency to treat patients with particular types of presenting problems (i.e., substance misuse and violence) moderated measurement-based matching may, preliminarily at least, provide evidence for further precision in conceptualizing personalized mental health care. Put simply, for therapists who consistently see patients with the most severe concerns and/or who consistently treat patients with certain types of problems, having them implement the match system could be especially important. Importantly, at least in the present sample, these two moderator findings seem closely related. That is, overall caseload-level severity was positively correlated with a higher caseload-level proportion of patients with primary substance misuse or violence. Therefore, although both of these interactions were statistically significant in our multiple moderator model, future research will need to tease apart whether caseload-level severity is actually a proxy for therapists’ tendency to specialize in treating problems that tend to be more severe (such as substance misuse and violence in this sample).

That being said, clinically, these results align with the broader therapist effects literature and may be one of those rare occurrences where patient-level and therapist-level effects are different sides of the very same coin. That is, a wealth of literature has demonstrated that the influence of the provider on patient outcomes is moderated by patient presenting problem severity—under the condition of working with patients with higher severity, some therapists become less effective, whereas others are consistently effective or may become even more effective (Coyne, 2024; Johns et al., 2019). One typical clinical implication of this patient-level moderator would be to try—to the extent possible—to connect patients with the highest severity levels to the most generally effective providers, as this would be one potent way to optimize their care. Indeed, a follow-up study to the Constantino et al. (2021) trial showed that patients with higher severity had even better outcomes when matched versus assigned as usual (Boswell et al., 2022). Moreover, in some ways, the current study also bore this out at the therapist level; that is, when these patients with higher severity (either in general or perhaps as a function of having a particular type of presenting problem that tends to be more severe) saw a given well-matched therapist who treated more of such individuals in the trial, they derived even greater use of matching than the average patient with lower presenting severity (who was working with a therapist who saw more of such individuals in their trial caseload).

Importantly, though, within the confines of the Constantino et al. (2021) RCT, any therapists who consistently saw more severe cases or a higher proportion of individuals with particular presenting problems (in what we have already described as a relatively small caseload) likely did so by chance. Thus, as therapist-level moderators of the match effect, it is plausible that caseload-level severity and specific problem proportion may be most clinically useful if they were indeed a therapist characteristic; for example, therapists who build their practice around *choosing* to see the most patients with primary substance misuse. With replication of the present results, it may be that these therapists are the ones who most need to adopt measurement-based matching and are the ones who implementation efforts can explicitly target. Alternatively, to optimize matching at a systems level, care networks may need to attend to *both* patient-level and therapist-level match moderators. For example, if a system did not wish to use matching for all new cases, they could rely on moderator research to take special care to always match certain individual cases (e.g., those with high severity baseline concerns; Boswell et al., 2022) and to always use the match algorithm when making assignments to certain therapists (e.g., those who want to see the most severe cases, or those who want to see individuals with primary substance misuse). However, as with the therapist effectiveness spread moderator, because these interactions were only significant for one outcome variable, caution is warranted when interpreting these effects and replication is needed before firm conclusions can be drawn. Additionally, future research may also identify other therapist characteristics that would make it especially important for their cases to be matched. As just one example, it could be that therapists who are most likely to “deskill” in the face of cases that are outside of their historical effectiveness wheelhouse are the ones who most need measurement-based matching to keep these skills intact and channeled toward patients who can most benefit from them.

This study had several limitations to note. First, it included a relatively small number of therapists, which could have limited our ability to detect significant interactive effects. Somewhat relatedly, this study also included a relatively small number of patients per therapist (*M* = 5.15), which could have limited our ability to reliably estimate therapists’ average outcomes with their match and CAU patients. In fact, simulation studies have revealed that the present patient and therapist sample sizes were at or slightly below the recommended minimum for having sufficient power (i.e., 80%) to detect interactions in multilevel data (e.g., Preacher et al., 2016). Therefore, it is possible that some of the null results observed in this study could stem from low power. Second, the naturalistic nature of the treatments meant that we had limited information about the specific interventions and practices therapists were using with trial patients. Future research should test whether specific in-session therapist behaviors and interventions explain therapist-level variability in the match effect. Third, two of the moderator variables were assessed using a study-specific measure with unknown psychometric properties (i.e., the TPS) and one moderator variable (effectiveness spread) was operationalized in a novel manner for the purposes of this study. The decision to adopt a dichotomous variable (*high* versus *low*) for the effectiveness spread factor was based, in part, on known therapist effectiveness classifications from the Constantino et al. (2021) trial. Alternative approaches to measuring and categorizing therapist effectiveness spread may be possible, and important, in larger therapist samples with greater effectiveness classification variability.

Fourth, measuring ROM usage with a self-report measure could have biased by social desirability and acquiescence. Finally, the patient and therapist subsamples in this study had relatively limited racial/ethnic diversity, which could limit the generalizability of the results. Limitations aside, the present results preliminarily point to three variables (therapist effectiveness spread, caseload-level patient presenting severity, caseload-level proportion of patients with primary violence or substance misuse) that explained some of the previously known therapist-level variability in the match effect, which provides some (though certainly as yet incomplete) insight into how patient-centered personalized care could be even more precise by also tailoring an evidence-based case-assignment system to the provider.

## Supplementary Information

Below is the link to the electronic supplementary material.

Supplementary file1 (DOCX 31 kb)

## Footnotes

1 As noted, a neutral classification is not inherently negative, as it can mean a therapist helps their average patient in a given problem domain improve to roughly the expected degree (Constantino et al., 2021). However, to the extent a patient believes a therapist is *highly* effective at treating their presenting problem, such an outcome may still be somewhat misattuned to the patient’s expectation.

2 See Supplemental Table 1 for descriptive statistics for each of the 12 domain-specific problems on the TOP at baseline by case assignment condition.

3 Notably, once assigned to the match condition, the level of matching was not randomly determined. Rather, from a shortlist of clinicians who were empirically matched at any level, a project coordinator worked their way through the list, from highest to lowest match level, to assign a therapist who met other necessary conditions for a given patient (e.g., had an opening, accepted their insurance; Constantino et al., 2021).

4 The remaining 36.1% of CAU patients were empirically unmatched.

5 Although more complex outcome change patterns (e.g., quadratic, cubic) are common in psychotherapy data, due to the variability in treatment lengths, many patients did not have enough timepoints to inform such complex trajectories. Therefore, consistent with the primary outcome paper for this trial (Constantino et al., 2021), we focused only on linear outcome change.

6 Due to variability in treatment lengths within this trial, we conducted a sensitivity analysis for the therapist effectiveness spread moderator with time centered at week 11 (the average treatment length in the sample). The cross-level interactive effect remained significant for both weekly global distress reduction and global distress level at week 11 (*p*s < .05). Full results available upon request of first author.
