# Supplementary material for: Therapist-Level Moderators of Patient-Therapist Match Effectiveness in Community Psychotherapy
Source: Adm Policy Ment Health. 2024 Apr 2;51(5):738–52. doi: 10.1007/s10488-024-01360-8 (PMC11379779; doi:10.1007/s10488-024-01360-8)
Supplement: Supplementary file 1 — Supplementary file1 (DOCX 31 kb) [file 10488_2024_1360_MOESM1_ESM.docx]

Online Supplement

Supplemental Table 1

*Domain-Specific Presenting Problem Severity by Condition (*N *= 206)*

|  | CAU (*n* = 108) | | Match (*n* = 98) | |
| --- | --- | --- | --- | --- |
| **Problem Domain** | *M* | *SD* | *M* | *SD* |
| Depression | 2.17 | 1.43 | 2.26 | 1.40 |
| Psychosis | 0.92 | 1.57 | 0.86 | 1.71 |
| Sleep | 0.96 | 1.43 | 0.94 | 1.59 |
| Suicidality | 1.11 | 1.89 | 1.05 | 1.92 |
| Violence | -0.08 | 0.70 | 0.27 | 1.95 |
| Manic | -0.33 | 0.64 | -0.12 | 0.83 |
| Substance Use | 1.62 | 3.50 | 1.49 | 2.87 |
| Work Functioning | 0.40 | 1.06 | 0.34 | 1.16 |
| Social Confidence | 0.65 | 1.51 | 1.15 | 1.55 |
| QOL | 2.26 | 1.06 | 2.21 | 1.10 |
| Sexual Functioning | 0.54 | 1.25 | 0.77 | 1.62 |
| Panic/anxiety | 1.23 | 1.78 | 1.23 | 1.69 |

*Note.* CAU = case assignment as usual; QOL = quality of life. For these TOP-based problem domain scores, a score of 0 represents the mean level of domain-specific impairment shown by non–treatment-seeking individuals in the community; a score of ±1 represents a domain-specific symptomatic/functional impairment level that is ±1 *SD* higher/lower than the community mean.

Multilevel Equation for Therapist-level Moderator Models

**Level-1 Model**

Outcome*_ijk_* = *π_0jk_* + *π_1jk_**(*Week17_ijk_*) + *e_ijk_*

**Level-2 Model**

*π_0jk_* = *β_00k_* + *β_01k_**(*Match_jk_*) + *β_02k_**(*patient-level severity_jk_*) + *r_0jk_*
*π_1jk_* = *β_10k_* + *β_11k_**(*Match_jk_*) + *β_12k_**(*patient-level severity_jk_*) + *r_1jk_*

**Level-3 Model**

*β_00k_* = *γ_000_* + *γ_001_*(*Moderator_k_*) + *γ_002_*(*Caseload-level severity_k_*) + *γ_003_*(*Caseload-level proportion*

*SU/V_k_*) + *u_00k_*
*β_01k_* = *γ_010_* + *γ_011_*(*Moderator_k_*) + *γ_012_*(*Caseload-level severity_k_*) + *γ_013_*(*Caseload-level proportion*

*SU/V_k_*) + *u_01k_*

*β_02k_* = *γ_020_*

*β_10k_* = *γ_100_* + *γ_101_*(*Moderator_k_*) + *γ_102_*(*Caseload-level severity_k_*) + *γ_103_*(*Caseload-level proportion*

*SU/V_k_*) + *u_10k_*

*β_11k_* = *γ_110_* + *γ_111_*(*Moderator_k_*) + *γ_112_*(*Caseload-level severity_k_*) + *γ_113_*(*Caseload-level proportion*

*SU/V_k_*) + *u_11k_*

*β_12k_* = *γ_120_*

Briefly, at level 1, the relevant patient outcome (i.e., the TOP total score or SCL-10 total score) was regressed on time in weeks, which was centered at week 17. Therefore, at level 2, the intercept (*π_0jk_*) represented the level of impairment severity at week 17 (hereafter “posttreatment”) for patient *j* who was treated by therapist *k* and the slope (*π_1jk_*) represented weekly rate of impairment change for patient *j* who was treated by therapist *k*. These two parameters were then predicted by match condition (*β_01k_*, *β_11k_*) and patient-level differences in presenting distress severity (*β_02k_*, *β_12k_*). At level 3, these coefficients dropped down to become the outcome variables, which were predicted by the relevant therapist-level moderator variable, therapist-level differences in their average patient’s presenting distress severity, and therapist-level differences in the proportion of patients with a primary presenting problem of substance use or violence (SU/V).

The fixed effects of interest represented: the main effect of the relevant moderator variable on posttreatment outcome level (*γ_001_*); the main effect of caseload-level severity on posttreatment outcome level (*γ_002_*); the main effect of caseload-level proportion of patients with a primary problem of SU/V (*γ_003_*); the effect of the relevant moderator on the match condition-posttreatment outcome level slope (i.e., cross-level interaction; *γ_011_*); the effect of caseload-level severity on the match condition-posttreatment outcome level slope (i.e., cross-level interaction; *γ_012_*); the effect of caseload-level proportion of patients with a primary problem of SU/V on the match condition-posttreatment outcome level slope (i.e., cross-level interaction; *γ_013_*); the average association between presenting patient-level severity and posttreatment outcome level (*γ_020_*); the main effect of the relevant moderator variable on weekly change in outcome (*γ_101_*); the main effect of caseload-level severity on weekly change in outcome (*γ_102_*); the main effect of caseload-level proportion of patients with a primary problem of SU/V on weekly change in outcome (*γ_103_*); the effect of the relevant moderator on the match condition-weekly outcome change slope (i.e., cross-level interaction; *γ_111_*); the effect of caseload-level severity on the match condition-weekly outcome change slope (i.e., cross-level interaction; *γ_112_*); the effect of caseload-level proportion of patients with a primary problem of SU/V on the match condition-weekly outcome change slope (i.e., cross-level interaction; *γ_113_*); and the average association between presenting patient-level severity and weekly impairment change (*γ_120_*). Random effects allowed posttreatment impairment level and weekly impairment change to vary among patients (*r_0jk,_ r_1jk_*) and therapists (*u_00k,_ u_10k_*). Additionally, random effects also allowed the match effect on posttreatment impairment level (*u_01k_*) and weekly impairment change (*u_11k_*) to vary among therapists.

*Supplemental Figure 1*. Caseload-level proportion of patients with substance use or violence as their primary problem as a moderator the within-therapist match effect on impairment severity.

*Note.* SU/V = substance use or violence; TOP = Treatment Outcome Package. The outcome variable (depicted on the y-axis) represents the TOP total score. For this outcome, a score of 0 represents the mean level of impairment shown by non–treatment-seeking individuals in the community; a score of ±1 represents a symptomatic/functional impairment level that is ±1 *SD* higher/lower than the community mean. In the figure, black lines depict average outcomes for different subgroups of patients in the match condition, whereas the CAU condition is represented by gray lines. Solid lines depict average outcomes for therapists with a higher proportion of patients with primary substance use or violence in their caseload (+1 *SD* above the mean) and dashed lines represent average outcomes for therapists with a lower proportion of these patients in their caseloads (-1 *SD* below the mean).

*Supplemental Figure 2.* Caseload-level severity as a moderator the within-therapist match effect on impairment severity.

*Note.* TOP = Treatment Outcome Package. The outcome variable (depicted on the y-axis) represents the TOP total score. For the TOP total score, a score of 0 represents the mean level of impairment shown by non–treatment-seeking individuals in the community; a score of ±1 represents a symptomatic/functional impairment level that is ±1 *SD* higher/lower than the community mean. In the figure, black lines depict average outcomes for different subgroups of patients in the match condition, whereas the CAU condition is represented by gray lines. Solid lines depict average outcomes for therapists with higher caseload-level severity (+1 *SD* above the mean) and dashed lines represent average outcomes for therapists with lower caseload-level severity (-1 *SD* below the mean).
